# The Role of Lubricin, Irisin and Exercise in the Prevention and Treatment of Osteoarthritis

**DOI:** 10.3390/ijms24065126

**Published:** 2023-03-07

**Authors:** Federico Roggio, Luca Petrigna, Bruno Trovato, Michelino Di Rosa, Giuseppe Musumeci

**Affiliations:** 1 Department of Biomedical and Biotechnological Sciences, Section of Anatomy, Histology and Movement Science, School of Medicine, University of Catania, Via S. Sofia 97, 95123 Catania, Italy; 2 Sport and Exercise Sciences Research Unit, Department of Psychology, Educational Science and Human Movement, University of Palermo, Via Giovanni Pascoli 6, 90144 Palermo, Italy; 3 Research Center on Motor Activities (CRAM), University of Catania, Via S. Sofia 97, 95123 Catania, Italy; 4 Department of Biology, Sbarro Institute for Cancer Research and Molecular Medicine, College of Science and Technology, Temple University, Philadelphia, PA 19122, USA

**Keywords:** lubricin, irisin, cartilage, exercise, joint motion, tribology, muscles, osteoarthritis

## Abstract

Osteoarthritis is a chronic degenerative musculoskeletal disease that worsens with age and is defined by pathological alterations in joint components. All clinical treatment recommendations for osteoarthritis promote exercise, although precise molecular pathways are unclear. The purpose of this study was to critically analyze the research on lubricin and irisin and how they relate to healthy and diseased joint tissue. Our research focused specifically on exercise strategies and offered new perspectives for future potential osteoarthritis treatment plans. Although lubricin and irisin have only recently been discovered, there is evidence that they have an impact on cartilage homeostasis. A crucial component of cartilage lubrication and integrity, lubricin is a surface-active mucinous glycoprotein released by the synovial joint. Its expression increases with joint movement. In healthy joints, lubricin molecules cover the cartilage surface to lubricate the boundary of the joint and inhibit protein and cell attachment. Patients with joint trauma, inflammatory arthritis, or genetically mediated lubricin deficiency, who do not produce enough lubricin to protect the articular cartilage, develop arthropathy. Irisin, sometimes known as the “sports hormone”, is a myokine secreted primarily by skeletal muscle. It is a physiologically active protein that can enter the circulation as an endocrine factor, and its synthesis and secretion are primarily triggered by exercise-induced muscle contraction. We searched PubMed, Web of Science, Google Scholar, and Scopus using the appropriate keywords to identify the most recent research. The studies considered advance our knowledge of the role that exercise plays in the fight against osteoarthritis, serve as a valuable resource, and support the advancement of osteoarthritis prevention and therapy.

## 1. Introduction

**Figure 1:**
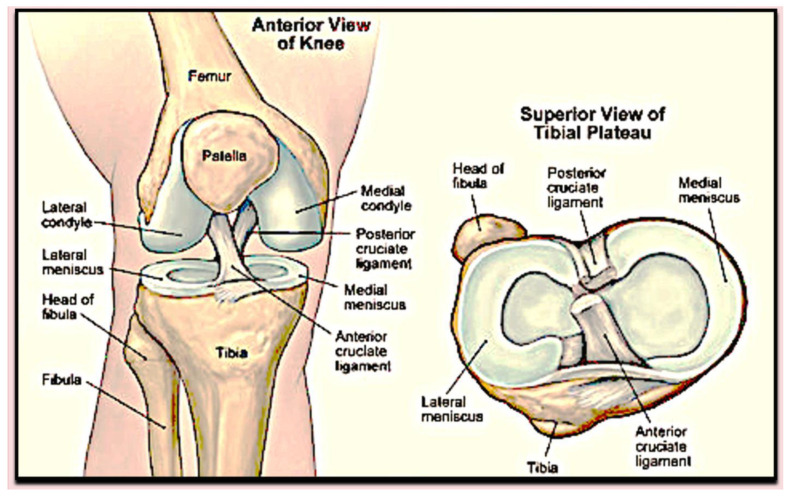
Anatomical structure of the knee joint.

The most common type of arthritis, osteoarthritis (OA) is a degenerative joint disease that primarily affects cartilage. Although there is an imbalance between anabolic and catabolic processes, the precise mechanism of cartilage degradation in OA remains unclear due to the complex interaction of genetic, environmental, metabolic, morphological, and biochemical factors [1,2]. It is one of the most prevalent worldwide health problems in orthopedics and can affect many joints; due to its complicated anatomical structure, the knee is the most affected joint (Figure 1). Large muscle groups and various ligaments stabilize this synovial joint [3], and the extracellular matrix gives cartilage tissue its distinct viscoelastic and compressive characteristics [4]. A thick layer of extracellular matrix (ECM) surrounding chondrocytes controls their activity and development, while preserving the biomechanical qualities of the tissue [5]. A specialized connective tissue, i.e., the synovium, maintains the fluid-filled space around the diarthrodial joints to provide a lubricating environment for the articulating surfaces [6]. Synovium produces particular lubricants for joint and paracrine chemicals that significantly affect the metabolism of articular cartilage [7,8]. In healthy joints, synovial lubrication reduces the friction coefficient of the joint, reducing heating and cartilage wear [9]. Degenerative joint disorders, such as OA, are considered to be triggered by a lack of synovial lubrication [10].

OA is characterized by osteophyte production and a gradual loss of articular cartilage, which causes chronic discomfort and functional limitations in affected joints. Numerous factors, including genetic predisposition, poor joint posture, aging, malnutrition, excessive use, hypomobility, and excessive body weight, can contribute to the development of OA. Traumatic events account for the majority of these causes [11]. Cartilage supports the bones to move smoothly against each other while minimizing friction. Unfortunately, the ability of articular cartilage to regenerate itself is restricted. The regenerated tissue that forms in response to cartilage loss is mainly fibrous, and its mechanical abilities are significantly reduced compared to healthy hyaline cartilage [12]. Since pro-inflammatory cytokines secreted by the inflamed synovium alter the activity of chondrocytes to create degradative enzymes, destroying cartilage, and preventing tissue repair and regeneration, changes in the normal function of type B synoviocytes are related to OA [10,13,14].

A significant issue in OA research is that the condition is rarely identified until joint abnormalities cause pain and are present on X-rays. Since early-stage OA generally does not manifest clinically, cartilage tissue from this stage of the disease is used for specific studies. For this reason, several animal models with OA have been developed to investigate the early signs of cartilage deterioration. Although the morphological and histological characteristics of OA are widely documented, the molecular causes are not yet well understood. Cartilage contains proteoglycans such as aggrecan and lubricating fluid composed of glycosaminoglycans such as hyaluronic acid and lubricin [15]. Lubricin, also known as the superficial zone protein, is a glycoprotein produced exclusively by synoviocytes and chondrocytes on the surface of the articular cartilage. There is evidence that lubricin, which provides the boundary lubrication of congruent articular surfaces under conditions of high contact pressure and almost zero sliding speed, is essential for normal joint function. The structural, functional, and biosynthetic characteristics of articular cartilage are significantly influenced by biomechanical factors [12]. The primary and secondary motions of the joint cause the contact area between the two surfaces to migrate during physiological joint loading. The migrating contact area is crucial to reduce the friction coefficient on the cartilage surface. Furthermore, lubricin expression has also been localized in the joint cavity. Numerous research studies have examined how lubricin affects joint disorders, namely OA, to stop cartilage wear and synovial cell adhesion and proliferation [11]. Recombinant lubricin therapy has also been shown in several trials to preserve cartilage in the articular region and stop the progression of OA in an animal model [16]. This implies that lubricin is crucial for the physiological maintenance of cartilage and may significantly affect the treatment or prevention of joint diseases. It could provide new information on how OA begins and progresses, serving as the biological foundation for future efforts in medical therapy to maintain tissue function and stop further cartilage damage.

Physical inactivity is the fourth most common cause of morbidity and mortality worldwide [17]. Numerous common chronic diseases, including OA, can be effectively managed with regular exercise. Myokine irisin, secreted by skeletal muscle in response to exercise stimulation and involved in resistance to aging, explains the protective mechanism of exercise in bone and cartilage tissues [18]. Irisin has been shown to improve the mechanical support of cartilage by subchondral bone, promote osteoblast differentiation and proliferation in the bone, and increase bone density and quality. On the other hand, irisin fosters chondrocyte proliferation, inhibits chondrocyte apoptosis, reduces the chondrocyte secretion of inflammatory factors and matrix metalloproteinases, and increases the stability of the extrachondral matrix. These aspects have great potential to offer further new insights into potential techniques in the treatment of osteoarthritis [19]. This narrative review will address recent publications in the literature on lubricin and irisin and their participation in healthy and diseased joint tissues. 

## 2. Molecular Mechanism of Lubricin and Cartilage 

### 2.1. Lubricin

Lubricin was originally identified as a lubricating glycoprotein in synovial fluid (SF), specifically synthesized and expressed by superficial zone articular chondrocytes. It is acknowledged to have a significant protective effect by lowering the friction coefficient on the surface of the articular cartilage, inhibiting synovial cell adhesion and proliferation, and preventing cartilage wear [20]. Lubricin is also known as the superficial zone protein, originally isolated and purified from the culture medium of explants derived from the superficial zone of bovine articular cartilage [20]. The joint lubrication system is known to include lubricin [21] and other components such as surface active phospholipids and hyaluronic acid (HA). The formation of a disulfide bond via the mismatched cysteine residue close to the C-terminus may be used to maintain lubricin adhesion to the articular surface [22]. According to measurements obtained using an electron microscope, the lubricin molecule is a partially stretched, flexible rod that, in solution, occupies less space than would be predicted by its structural makeup. This distinctive quality of the lubricin molecule may contribute to the molecule’s capacity to lubricate boundaries. 

The B-somatomedin and hemopexin-like domains are present in the 1404-amino-acid sequence of lubricin, which has been proposed to control the complement and coagulation systems, facilitate the adhesion of the extracellular matrix, and promote cell attachment and proliferation [22]. Purified hemopexin interacts with HA, indicating that lubricin binding to HA located at or close to the articular surface may also be mediated by the hemopexin-like domain [21]. 

### 2.2. Lubricin and Cartilage

Numerous cartilage problems have also been associated with the depletion of lubricin function [23]. The expression and/or abundance of secreted lubricin is significantly reduced when synoviocytes, chondrocytes, and cartilage explants are exposed to pro-inflammatory cytokines such as interleukin-1 and tumor necrosis factor-alpha, with corresponding changes in the amounts of lubricin associated with cartilage [6]. On the other hand, the production, secretion, and association of cartilage boundary lubricin are markedly enhanced by exposure to beta transforming growth factor [16]. Lubricin is essential for the physiology of the articular joints, and its loss or accumulation can contribute to the pathology of OA. Due to this, current research has shown that recombinant lubricin plays a role in chondroprotection in an animal model of OA, raising the possibility of using recombinant lubricin molecules in new therapeutic strategies for the treatment of OA and related cartilage abnormalities [16]. Therefore, finding new, potentially effective biotherapeutic methods to treat OA is of great interest. Lubricin expression decreases with age and throughout OA, and the lubricin gene has differential expression in rheumatoid arthritis (RA) and OA synovium, suggesting that it may play a role in the pathophysiology of both diseases. Two important “boundary lubricants” that show low friction and protect surfaces from wear are HA and lubricin, both used as joint lubricants [24]. HA and lubricin are necessary for the proper operation of the entire lubrication system. Both lubricin and HA have other purposes in the joint, in addition to lubrication, such as regulating synovial cell adhesion and proliferation. In a rat model of OA caused by meniscectomy and medial collateral ligament, researchers found that the intraarticular injection of recombinant lubricin prevented cartilage deterioration [16]. Furthermore, lubricin protects against cartilage superficial zone cell loss, collagen deterioration, and glycosaminoglycan depletion [25]. The researchers tested isolated lubricin from human synovial fluid (SF), compared it to HA, and mixed the two in a rat model of OA [26]. These findings supported the ability of lubricin to provide protection, and the inclusion of exogenous HA, which did not have protective effects, improved the therapeutic benefit [25,26]. These investigations offer a promising opportunity for lubricants to serve as therapeutic targets in post-traumatic arthritis, which is associated with a severe shortage of lubricants [27]. They also provide new information on the function of joint lubricants as possible therapeutics for OA. 

## 3. Molecular Mechanism of Irisin and Cartilage

### 3.1. Irisin and the Bone–Muscle Cross-Talk

Irisin is a myokine released in the bloodstream from the skeletal muscles, which is triggered by muscle contraction and cold exposure from the stimulation of peroxisome proliferator-activated receptor gamma coactivator 1-alpha (PGC-1α) [28,29]. This myokine is composed of 112 aa residues and it is a cleavage protein of fibronectin type III domain 5 (FNDC5); moreover, FNDC5 is converted to irisin after physical activity is performed, causing also the browning of white adipose tissue both in mice and humans [30]. In a previous study published in *Nature* in 2012 by Boström et al. [31], it was identified that irisin promotes the trans-differentiation of white adipose tissue into its brown type, playing an important role also in regulating thermogenesis during physical activity. Nowadays, irisin is considered to have a crucial role both in bone and muscle metabolism. In the muscle tissue, it was seen that irisin increased the expression of mitochondrial factors that bring an increment in mitochondrial content and oxygen consumption [32]. It was found in mice that irisin promotes the differentiation of bone marrow stromal cells in osteoblasts, and also enhances osteoblastic parameters such as the number of alkaline phosphatase colonies and the gene levels of type I collagen [33]. A study by Zhang et al. [18] in mice found that irisin promotes osteoblast differentiation through the Wnt/β-catenin pathway in osteoblastic MC3T3-E1 cells, with irisin inhibiting osteoclast differentiation. Thus, in the complex bone–muscle cross-talk, irisin may be considered as a muscle-derived bone anabolic factor [34]. The roles of irisin in this physiological process between muscle and bone driven by mechanical stresses are still not fully understood yet. Therefore, further studies investigating irisin in humans are needed to gain better knowledge of its functioning. 

### 3.2. Irisin and Cartilage

By controlling chondrocyte proliferation and apoptosis and preserving the integrity of the extrachondral matrix, irisin can directly affect the onset of osteoarthritis. The inflammatory process of OA causes the death of chondrocytes and the breakdown of the extracellular matrix [35]. The first investigation of the relationship between irisin concentrations and osteoarthritis included blood and SF samples from patients with knee OA. Irisin concentrations in serum and SF decreased with increasing Kellgren–Lawrence radiological classification, and the results showed a negative correlation between irisin concentrations and OA severity on the imaging of OA [36,37]. With a decrease in irisin concentration, the expression of C-reactive protein (CRP) in serum increased [38]. Serum levels of irisin are influenced by different factors, such as gender, body mass index, muscle mass, age, and training [39]. In cultured three-dimensional human osteoarthritic chondrocytes, r-irisin enhanced type II collagen levels while reducing the expression of the cartilage ossification marker type X collagen. Administration of r-irisin reduced the phosphorylation levels of p38, Akt, JNK, and NF-κB but not phosphorylated ERK in chondrocytes for a short period, in contrast to its findings in osteoblasts [40]. These findings suggest that irisin signaling in osteoblasts and chondrocytes may be cellularly specific. Irisin decreased the production of the inflammatory mediators IL-1 and IL-6, blocked the death of chondrocytes, downregulated the metalloproteinases MMP-1 and MMP-13, preserved the protective role of the extracellular matrix in cartilage, and encouraged chondrocyte proliferation [40]. Furthermore, researchers showed that irisin injection improved gait in mice that underwent surgical destabilization of the medial meniscus (DMM) and prevented the loss of autophagic markers Atg4 and Atg12, as well as p62, caused by IL-1β. Irisin strengthens the membrane potential of chondrocytes and mitochondrial biogenesis to suppress chondrocyte apoptosis and enhance the formation of the extracellular matrix, protecting articular cartilage and delaying the onset of OA [41]. On the other hand, there are conflicting findings on the capacity of irisin to promote chondrocyte proliferation. Other researchers found that in mice with anterior cruciate ligament transection (ACLT), there was a substantial increase in the proportion of hyaline cartilage and a decrease in the calcification of the tibial cartilage at the knee joint. The findings revealed that the proliferative capacity of the chondrocytes after receiving irisin intravenously did not change, as seen in an in vitro pulling assay that mimicked exercise. As a result, improving the microstructure of the subchondral bone and lowering bone cell apoptosis were the main ways in which irisin’s pain-relieving effects on OA were achieved [42]. Scientists cultured and studied KI to KO irisin in transgenic mice to better understand the function of irisin in cartilage tissue [37]. In contrast to KI mice with the intraarticular injection of irisin, irisin knockout mice showed more severe OA symptoms after modeling DMM in vivo. These KI mice showed strong resistance to DMM-induced OA development. In studies using primary chondrocytes cultured in vitro, r-irisin restored the proliferative capacity of KO cells and significantly increased cell proliferation in irisin KI mice in both normal and IL-1β-induced inflammatory states. In contrast, mouse-derived KO chondrocytes had a decreased proliferative capacity. Irisin increased the expression of COL2a1, aggrecan, and SOX9, while negatively regulating the production of inflammatory mediators and factors in chondrocytes. These results demonstrate how irisin encourages chondrocyte growth [19]. However, the authors postulated that variations in vitro mechanical pull parameters and the types of cell culture used in the various investigations may have influenced the variances in the results of these investigations. Primary chondrocytes grown in three dimensions can more accurately mimic irisin’s effects in vivo. 

In OA, chondrocytes and the Wnt/β-catenin and NF-κB signaling pathways are inhibited by the high expression of irisin. A metabolic imbalance between anabolic and catabolic substances produced by chondrocytes causes the cartilage to break down and be destroyed. This is a significant factor in chondrocyte death and the deterioration of extrachondral processes, which eventually result in OA [43,44]. In a recent study, researchers verified that Sirt3, a signaling molecule strongly linked to the activation of the Wnt pathway, exerts a protective impact on chondrocyte mitochondria by blocking apoptosis and reducing oxidative damage in inflammatory chondrocytes [41]. To replicate OA in vitro, the researchers used IL-1β to generate osteosarcoma cells. The results demonstrated that the administration of irisin dramatically reduced the levels of WnT-1 and β-catenin protein and mRNA. The impact of LiCl induction on β-catenin was also dramatically reduced by irisin intervention. The amount of type II collagen protein was reversed in IL-1β-induced osteosarcoma cells, while MMP-13 and other metal matrix proteases were expressed in cartilage tissues. Irisin has shown potent anti-inflammatory properties [45]. On the other hand, irisin reversed the IL-1β-induced IkBa expression and p65 phosphorylation levels in osteosarcoma cells, as shown by lower TNF-α expression in chondrocytes cultivated in vitro [19]. Irisin reduced the activity of the NF-κB pathway and suppressed cytoplasmic p-p65 in chondrocytes, showing potent anti-inflammatory properties [45]. Significant pro-inflammatory programmed cell death, known as chondrocyte scorch death, was ameliorated in vitro by increased irisin concentrations in morphological studies [46]. In another recent study, irisin pretreatment blocked the IL-1β-induced NF-κB/p65 nuclear translocation of NF-κB/p65 and increased the expression of chondrocyte-specific collagen II while inhibiting nod-like receptor protein-3 (NLRP3)/caspase-1. Irisin improved OA by preventing the activation of the PI3K/Akt/NF-κB cascade response and decreasing the inflammation-induced scorching of chondrocytes, according to the results of biochemical analysis and biochemical indices [46]. Irisin had a protective effect on cartilage by preventing the Wnt/β-catenin and NF-κB signaling pathways, decreasing the expression of matrix metalloproteinases, improving the stability of the extracellular matrix, reducing inflammatory factors in chondrocytes, inhibiting apoptosis, and encouraging chondrocyte proliferation [47].

## 4. Exercise, Lubricin, Irisin, and Cartilage in Osteoarthritis 

### 4.1. The Role of Physical Activity in Cartilage Degeneration

**Figure 2:**
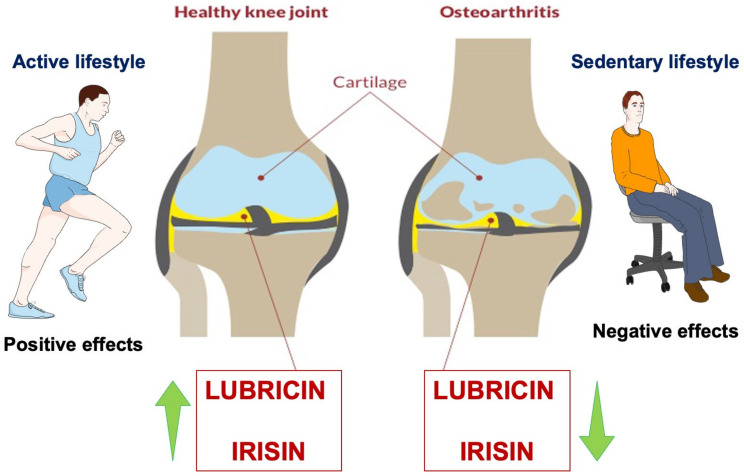
Irisin and lubricin can act directly on cartilage tissue and influence the disease process of OA by inhibiting inflammation and promoting joint lubrication and tribology. The green arrows represent the increase or decrease of it depending on the lifestyle.

Authors have investigated how moderate physical activity, typical joint loading, and mechanical stimulation in elderly rats improved lubrication and prevented cartilage degeneration, promoting lubricin synthesis in SF, compared to unexercised adult rats, to understand better the potential of exercise in lubricin expression and tribology [48]. Exercise promotes joint motion, which causes SF and chondrocytes to produce more lubricant, improving the lubrication of articular surfaces (Figure 2). With age and other factors, including diet and osteoporotic disease, this preventive lubrication mechanism helps to delay the onset of OA [49]. These findings also lend credence to the idea that physical exercise may be used as a preventive measure against OA and as a natural remedy for cartilage problems as people age. The exercise program must be mild to moderate and primarily “adapted”, “personalized,” or “tailored”, since the intensity of the activity will depend on how well the individual can tolerate it (their unique physical characteristics/biotype) [50]. Prescriptions for preventive exercise training should be written similarly to those for medications. It should be prescribed in the same way as pharmacological treatment, deciding on the “dosage” and “formulation” for each patient. The “dosage” is determined to achieve a particular degree of effectiveness that reduces or eliminates symptoms without having harmful consequences. It is crucial to provide customized guidelines or “formulations” for each person experiencing or at risk of such challenges [50]. Exercise therapy may decrease cytokine expression and related gene expression and inhibit inflammatory factor-mediated cartilage degradation through the synthesis of IL-4, IL-10, and irisin by type A synoviocytes, thus effectively blocking cartilage damage. 

### 4.2. Lubricin and Exercise in Healthy Cartilage and OA

Moderate physical activity, as a nonsurgical and non-pharmacological strategy, could restore normal synoviocyte function and, as a result, allow cartilage preservation in the early stages of OA, delaying the formation of visible OA and, ultimately, delaying the need for joint replacement. Walking, performing chores around the house, slow running, swimming, biking, and using elliptical machines, among other activities, may protect joints from the damaging effects of excessive repetitive joint use and replace glycoproteins lost during periods of inactivity, improving clinical outcomes and joint lubrification [10,51]. Encouraging lubricin production and its elevation in synovial fluid, moderate physical activity, and typical mechanical joint loading increase the tribology and lubricative quality of the articular cartilage [52,53,54,55], preventing cartilage degeneration. However, longer-term in vitro, in vivo, and clinical studies are required to fully understand the precise morpho-molecular function of lubricin in the context of cartilage aging, damage, and degeneration, although this contribution emphasizes the advantages of moderate exercise for articular cartilage.

### 4.3. Irisin and Exercise in Healthy Cartilage and OA

The preventive mechanism of exercise in cartilage tissues to prevent and manage OA was better elucidated when irisin was discovered, and it was described as a myokine released by skeletal muscle in response to exercise stimulation, implicated in resistance to aging [47,56,57]. Therefore, some research investigated the possibility of modulating irisin through physical activity. Intensive exercise can raise serum levels and Tsuchiya et al. [58] showed that high-intensity physical activity was more suitable to enhance serum levels compared to low-intensity exercise performed in the same individuals on different days. Furthermore, exercise has the benefit of reducing the inflammatory state of OA, and there is a link between inflammation resistance and irisin expression. Recent research found that older women with osteoarthritis and sarcopenia who exercised at moderate intensity had significantly higher blood levels of anti-inflammatory proteins irisin and IL-10, which reduced the proinflammatory TNF-α production and improved the OA index [59]. In recent years, other studies on the protective effects of irisin on cartilage tissue have been reported. A study in rhythmic gymnasts evidenced how serum levels of irisin were positively associated with energy expenditure positively compared to untrained controls [60]. Recent research evaluated the impacts of low, moderate, and high treadmill activity on the effects on irisin concentration levels in the synovial fluid of SD rats [46]. This study showed that exercise of the appropriate intensity reduced cartilage damage in a rat model and achieved therapeutic effects in OA. It also showed that exercise at the appropriate intensity significantly increased the irisin concentration in the synovial fluid, reduced the inflammation of chondrocytes, and reduced cartilage damage. However, high-intensity exercise may result in excessive mechanical stimulation, which can cause damage that negates the beneficial effects of higher irisin concentrations and accelerates the development of OA. The main results have been summarized in Table 1.

## 5. Perspectives

**Figure 3:**
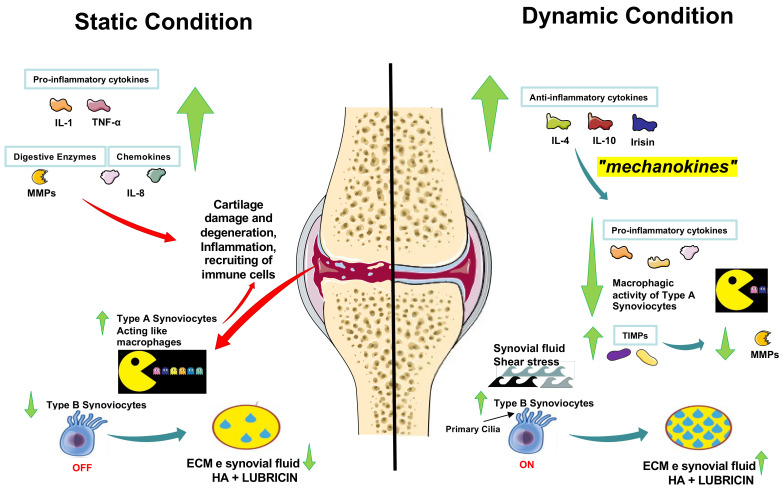
Hypothetical mechanisms of action concerning possible immunological/molecular developments in the OA process within the cellular components of the synovial membrane. OA cartilage during static condition. Chronology of events: 1. Type A cartilage damage phagocytosis of cartilage damage; 2. Matrix metalloproteinase (MMP) and chemokine release; 3. Immunological synapse; 4. Release of pro-inflammatory cytokines; 5. Synoviocyte type B transformation in fibroblast-like cells; 6. Reduction in the synthesis of type B synoviocyte matrix (HA + lubricin). OA cartilage during dynamic condition. Chronology of events: 1. Synoviocytes type B primary cilia stimulation by shear stress movement of the synovial fluid; 2. Tissue inhibitors of metalloproteinase (TIMP) production to counteract MMPs; 3. Release of anti-inflammatory cytokines (called mechanokines) (irisin); 4. Type A synoviocyte modulation; 5. Pro-inflammatory cytokine reduction; 6. Synoviocyte type B matrix synthesis (HA + lubricin) increased. The green arrows represent the increase or decrease of it depending on the lifestyle.

The role and mechanisms of irisin in bone and cartilage tissues have received a great deal of attention in the treatment of OA using exercise. We support the idea that once irisin is activated through physical activity (Figure 3), it plays an important role in protecting the articular cartilage [19,46] and preventing OA [42,52], as shown already in previous studies. Based on the assumptions made in this review, irisin may be a novel marker signal and therapeutic target in this condition. However, both lubricin and irisin are exercise-induced molecules; rather than considering the general changes due to different physical stimuli, future studies should investigate the different stress levels in the articular joint, because the compression of the joint may be the key point to focus on in order to understand the threshold of positive or negative effects on the joint. For example, while Orellana et al. [62] observed that irisin levels were negatively related to exercise levels in the studied population, Liu et al. [63] measured increased serum irisin after static strength training in rats. Although the relationship between physical activity and irisin is still unclear, tailored physical activity programs will aid research on the correct dose of physical activity to foster a protection mechanism against OA degeneration. More research into the mechanisms governing the effects on cartilage metabolism and the effects of exercise form and intensity on the induction of irisin expression will help to develop more effective osteoarthritis prevention and treatment options.

## 6. Conclusions

**Figure 4:**
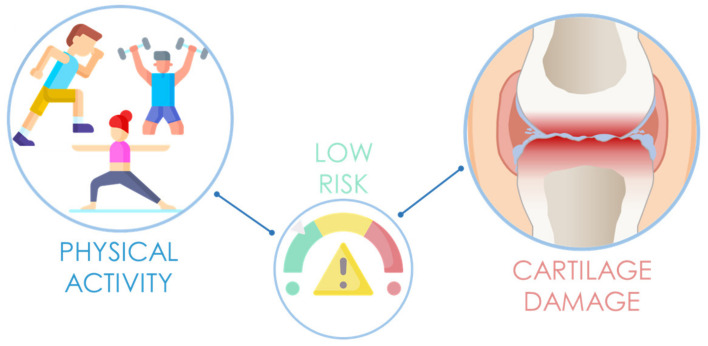
The benefits of moderate and adapted physical activity for the prophylaxis and treatment of OA.

The information provided above suggests that irisin and lubricin can act directly on cartilage tissue and influence the disease process of OA by inhibiting inflammation and promoting joint lubrication and tribology. With this review, we can confirm that lubricin and irisin are fundamental biomarkers for the detection of chondrocytes affected by OA and their important role during exercise. This knowledge may help to develop novel, innovative, and preventive strategies to slow or stop the aging process and the development of OA in joint tissues due to moderate physical activity. Today, the benefits arising from moderate and adapted physical activity for the prophylaxis and treatment of OA are well established in the scientific community, as many international medical societies have recently recommended them in preventing joint diseases (Figure 4). For some chronic diseases, such as OA, well-planned exercise interventions and a healthy diet are at least as effective as drug therapy.

## Figures and Tables

**Table 1:** Main findings of the studies regarding irisin, lubricin, and physical activity in osteoarthritis.

Study	Sample	Investigation	Main Markers Evaluated	Conclusions
Castrogiovanni et al., 2019 [10]	Male Wistar rats divided into groups: control, moderate exercise, ACLT and early OA, ACLT early OA and moderate exercise	Effects of 2 weeks post ACLT, 25 min of moderate running 3 times per week, for 12 weeks, on cartilage-related biomarkers	Synovium, IL-1β, IL-4, IL-6, IL-10, TNF-α, MMP-13, lubricin	Moderate physical activity supported joint tribology, decreased OA-related biomarkers and increased the chondroprotective ones.
Szychlinska et al., 2016 [15]	Wistar outbred rats divided into groups: control non-operated group, control operated without ACTL, OA rats with ACTL	Investigation of CHI3L and lubricin and their association with OA	Sections of articular cartilage, radiographs, CHI3L and lubricin	Dysregulation of CHI3L and lubricin could be related to OA development, especially when a KL grade of 2/3 is present
Zhang et al., 2017 [18]	Wild-type C57BL/6J and APN-KO mice divided into groups: injected with irisin, injected with saline, injected with lentiviral FNDC5, control EGFP	Effects of 2 weeks of voluntary wheel running on cartilage	FNDC5/irisin, PGC1α, UCP1	FNDC5/irisin expression is enhanced by 2 weeks of voluntary wheel running at mRNA and protein levels in mice
Li et al., 2021 [19]	Irisin over-expressing mice divided into groups and tissue from humans: mice ACLT with PBS, mice ACLT with irisin, deceased human donors	In vitro mechanistic experiment to understand the functioning of irisin	Irisin, IL-1B, IL-6, TNF-α, COX2, MMP-13	Irisin is important in the development of cartilage and in OA pathogenesis; moreover, irisin could represent a promising therapeutic target in OA
Rhee et al., 2005 [23]	Wild-type and lubricin mutant mice	Clinical, radiological and histological characterization and immunohistochemical analysis of lubricin mutant mice to understand the multiple functioning of lubricin during development	Lubricin, synoviocites	Lubricin is fundamental to prevent the deposition of protein onto cartilage from SF, and to inhibit the adhesion of synovial cell to cartilage
Jay et al., 2010 [25]	Lewis rats divided into groups: unilateral ACLT, control	Rats were injected with PBS, HSL, rhPRG4 or HSFL twice a week, 7 days after injury, to investigate the degeneration of cartilage	Safranin O stained tibial plateau cartilage, PBS, HSL, rhPRG4 or HSFL, urinary C-terminal cross-linked telopeptide	Regardless of the type of lubricin injection performed, the cartilage degeneration after ACTL was reduced. Intraarticular lubricin injection can delay the degeneration of cartilage
Teeple et al., 2011 [26]	Male Lewis rats with ACLT divided into groups based on injection type: LUB, PBS, HA, LUB + HA	Injection of LUB, PBS, HA, LUB+HA to understand if lubricin and/or hyaluronic acid supplementation can reduce cartilage damage in mice with ACLT	CTXII, IL-1β, TNF-α, lubricin, and sGAG, KL grade	LUB+HA supplementation had positive effects on the cartilage of ACLT rats; conversely, HA alone was not effective
Mao et al., 2016 [38]	Humans with and without knee OA	Investigation of the levels of irisin and its correlation with OA through enzyme-linked immunosorbent assay	C-reactive protein, irisin	Authors found a negative correlation between the levels of irisin in SF and OA classified with KL grade
Löffler et al., 2015 [39]	Humans divided into groups depending on the type of exercise: acute maximal (15 min), short-term acute (30 min), mid-term moderate (6 weeks), long-term low-grade (1 year follow-up)	People were tested for serum irisin levels after performing physical activity of different types and for different times (from 15 min to 1 year) to understand how irisin is influenced by exercise	Irisin, glucose, insulin, triglycerides, cholesterol, testosterone	Irisin’s serum levels are positively correlated with muscle mass. Interestingly, serum irisin levels rose only in the short-term exercise group
Vadalà et al., 2020 [40]	Tissue from human patients with OA was divided into groups: cells exposed to PBS and cells exposed to human recombinant irisin	Investigation of the effects of irisin on hOAC through RNA extraction and gene expression analysis, protein extraction and Western blot analysis	Irisin, glycosaminoglycans, collagen type II, ERK, MAPK, Akt, JNK, NF-κB, IL-1, IL-6, MMP-1, MMP-13, TIMP-1, TIMP-3	The cells exposed to irisin showed increased hOAC content, collagen II protein and gene expression and decreased type X collagen expression. In addition, irisin decreased the levels of IL-1, IL-6, MMP-1, MMP-13 gene expression; conversely, TIMP-1 and TIMP-3 levels increased
Wang et al., 2020 [41]	Tissue from human patients with OA and C57L/B6 mice DMM	Investigation of knee OA in humans an in DMM mice to understand the signaling pathway of FNDC5 (the precursor of irisin)	FNDC5, IL-1β, irisin	Irisin is protective against chondrocyte dysfunction
He et al., 2020 [42]	C57BL/6J mice divided into groups: sham operated, ACLT with saline, ACLT with irisin	Investigation of the possibility of using irisin in OA as a therapy	Irisin, ERK	Irisin may attenuate cartilage degeneration in mice by the inhibition of osteocyte apoptosis, activating the ERK pathway
Li et al., 2020 [45]	Chondrosarcoma cell line SW1353 from human	Western blotting and immunofluorescence analysis of SW1353 cells to understand the interaction of irisin, Wnt/β-catenin and NF-κB pathways	Irisin, IL-1β, MMP-13, NF-κB, collagen II	Irisin can reverse the interleukins’ effects in SW1353 cell line, suggesting that irisin may be protective for cartilage
Jia et al., 2022 [46]	Sprague-Dawley rats divided into groups: control, OA, OA + exercise	Investigation of the effects of physical activity on levels of irisin	Radiographs, irisin, IL-1β, MMP-13, ADAMTS-5	The mechanical stimulation given by moderate exercise protects against OA thanks to irisin. Irisin counterbalances the negative inflammatory effects of IL-1β by suppressing it
Musumeci et al., 2015 [48]	Wistar rats divided into groups: sedentary and physically active	8 weeks of physical activity in total, 2 weeks on treadmill with no inclination for 10 min 5 times per week, 6 weeks on treadmill with 3° slope for 20 min 5 times per week, to analyze the expression of lubricin as a marker of chondrocyte senescence	Lubricin	Moderate physical activity improved lubrication and prevented cartilage degeneration in old rats
Musumeci et al., 2013 [49]	Albino Wistar rats divided into: control, OA + prednisolone, prednisolone + treadmill, prednisolone + vibration exercise, prednisolone + vibration exercise + treadmill	Evaluating the effects of different types of physical activity on the expression of lubricin and caspase-3 after prednisolone injection	Lubricin, caspase-3, β tubulin, prednisolone	Exercise was beneficial for cartilage, probably through the expression of lubricin, which inhibits caspase-3, avoiding chondrocyte death
Roberts et al., 2016 [52]	Humans divided into groups: runners and cyclists	ELISA analysis to investigate serum lubricin and COMP, ultrasonography of femoral cartilage and how they respond to running and cycling	Lubricin, COMP, ultrasonographic assessment	Acute bouts of running or cycling elicit an increment in cartilage metabolism; moreover, COMP and lubricin levels significantly increased after exercise
Ni et al., 2012 [54]	Male Wistar rats divided into groups: control, low-intensity running, moderate-intensity running, high-intensity running	8 weeks of different types of treadmill training to highlight the differences in cartilage lubricin expression	Lubricin, TGF-β, PRG4	Low- or moderate-intensity running upregulates PRG4, causing an increase in lubricin. Conversely, excessive running led to a decrement in lubricin levels
Elsaid et al., 2012 [55]	Lewis rats divided into groups: 3-week post-ACLT, 3-week post-ACLT + exercise, 5-week post-ACLT + exercise + one intra-articular sham injection of PBS, 5-week post-ACLT + exercise + one intra-articular injection of purified human lubricin	Investigation of the effects of lubricin injection and physical activity in relationship with cartilage degeneration	Lubricin, uCTX II	Intraarticular lubricin injection ameliorated cartilage damage thanks to exercise and preserved the chondrocytes
Ravalli et al., 2022 [61]	Wistar outbred rats divided into groups: control, OA, OA + exercise, control + exercise	Rats subjected to physical activity on treadmill 3 times per week for 12 weeks with a progressive duration from 5 to 25 min, to evaluate ATX and lubricin in the process of cartilage homeostasis	ATX, lubricin	ATX may be involved in mild OA and moderate exercise reduced ATX in the femur; lubricin was found in significantly lower concentrations in OA, suggesting its use as a biochemical biomarker
Tsuchiya et al., 2014 [58]	Humans divided into groups: treadmill running at 40% of VO_2_max for 40 min, treadmill running at 80% of VO_2_max for 20 min	Blood sample collection before and post-exercise to investigate the fluctuations in irisin levels before and after exercise	VO_2_max, irisin, LDL, HDL	In this preliminary study, it is suggested that a single bout of acute running enhances irisin levels more than low-intensity exercise

ACTL = anterior cruciate ligament transection; OA = osteoarthritis; IL-1β = interleukin 1β; IL-4 = interleukin 4; IL-6 = interleukin 6; IL-10 = interleukin 10; TNF-α = tumor necrosis factor alpha; MMP-13 = matrix metallopeptidase 13; CHI3L = Chitinase 3-like protein 1; KL = Kellgren and Lawrence; EGFP = enhanced green fluorescent protein; COX2 = cyclo-ossigenase 2; SF = synovial fluid; PBS = phosphate-buffered saline; HSL = human synoviocyte lubricin; rhPRG4 = recombinant human proteoglycan 4; HSFL = human synovial fluid lubricin; HA = hyaluronic acid; LUB = purified human lubricin; sGAG = sulphate glycosaminoglycans; hOAC = osteoarthritic chondrocytes; ERK = p38/extracellular signal-regulated kinase; MAPK = mitogen-activated protein kinase; Akt = protein kinase B; JNK = c-Jun N-terminal kinase; NF-κB = nuclear factor kappa-light chain enhancer of activated B cells; TIMP = tissue inhibitor of metalloproteinase; DMM = destabilized medial meniscus; FNDC5 = fibronectin type III domain containing protein 5; ADAMTS-5 = metalloproteinase with thrombospondin motifs; COMP = cartilage oligomeric matrix protein; TGF-β = transforming growth factor beta; PRG4 = proteoglycan 4; ATX = autotaxin; LDL = low-density lipoprotein; HDL = high-density lipoprotein.

## Data Availability

Not applicable.
